# Detecting Photoactivatable Cre-mediated Gene Deletion Efficiency in *Escherichia coli*

**DOI:** 10.21769/BioProtoc.4685

**Published:** 2023-06-05

**Authors:** Yuta Koganezawa, Yuichi Wakamoto, Moritoshi Sato, Miki Umetani

**Affiliations:** 1 Department of Basic Science, Graduate School of Arts and Sciences, The University of Tokyo, Tokyo, Japan; 2 Research Center for Complex Systems Biology, The University of Tokyo, Tokyo, Japan; 3 Universal Biology Institute, The University of Tokyo, Tokyo, Japan; 4 Department of Life Sciences, Graduate School of Arts and Sciences, The University of Tokyo, Tokyo, Japan; 5 Kanagawa Institute of Industrial Science and Technology, Kanagawa, Japan

**Keywords:** Gene deletion, Genotype–phenotype mapping, Optogenetics, *Escherichia coli*, Antibiotic resistance

## Abstract

Gene deletion is one of the standard approaches in genetics to investigate the roles and functions of target genes. However, the influence of gene deletion on cellular phenotypes is usually analyzed sometime after the gene deletion was introduced. Such lags from gene deletion to phenotype evaluation could select only the fittest fraction of gene-deleted cells and hinder the detection of potentially diverse phenotypic consequences. Therefore, dynamic aspects of gene deletion, such as real-time propagation and compensation of deletion effects on cellular phenotypes, still need to be explored. To resolve this issue, we have recently introduced a new method that combines a photoactivatable Cre recombination system and microfluidic single-cell observation. This method enables us to induce gene deletion at desired timings in single bacterial cells and to monitor their dynamics for prolonged periods. Here, we detail the protocol for estimating the fractions of gene-deleted cells based on a batch-culture assay. The duration of blue light exposure significantly affects the fractions of gene-deleted cells. Therefore, gene-deleted and non-deleted cells can coexist in a cellular population by adjusting the duration of blue light exposure. Single-cell observations under such illumination conditions allow the comparison of temporal dynamics between gene-deleted and non-deleted cells and unravel phenotypic dynamics provoked by gene deletion.

## Background

Analyzing the phenotypes of gene-deleted cell strains or multicellular organisms is the cornerstone of genetics studies. The functional roles of various genes in each organism can be deduced from the phenotypic changes caused by their deletion. For systematically analyzing the effects of gene deletion, gene knock-out libraries are available for several model organisms (Jorgensen et al., 2002; Baba et al., 2006; Kim et al., 2010; White et al., 2013). For example, the Keio collection (Baba et al., 2006) is a single-gene knock-out library of *Escherichia coli* that has been used to infer the roles of genes in various biological phenomena, such as cellular growth and morphogenesis (Campos et al., 2018), antibiotic sensitivity (Tamae et al., 2008), and evolution (Lukačišinová et al., 2020).

However, the significance of each gene might be context dependent. Gene expression levels change dynamically depending on environmental conditions (Schmidt et al., 2016; Bhatia et al., 2022). In Koganezawa et al. (2022), we induced a Cre-mediated antibiotic resistance gene deletion with blue-light illumination in the presence of the antibiotic. The microfluidic technique revealed that a small fraction of the gene-deleted cells could continue their growth without other genetic changes, and the fraction varied depending on the history of antibiotic administration. These results indicate that the impact of gene deletion on cellular physiology can depend on historical conditions (Koganezawa et al., 2022). Therefore, revealing dynamic genotype–phenotype relationships is crucial to understand the functional roles of each gene in given biological contexts.

Optogenetic techniques are useful for analyzing dynamic genotype–phenotype relationships. In particular, the photoactivatable Cre (PA-Cre) system allows us to induce gene deletion at desired timings by blue light exposure (Kawano et al., 2016). The PA-Cre system consists of split Cre recombinase, CreC, and CreN, fused to p-Magnet (p-Mag) and n-Magnet (n-Mag) protein fragments, respectively (Kawano et al., 2016). p-Mag and n-Mag are derived from a fungal photoreceptor Vivid and form a dimer upon blue light illumination (Kawano et al., 2015). Therefore, when these components of the PA-Cre system are expressed in the cytoplasm of bacterial cells, blue light illumination can induce the deletion of a target gene sandwiched between the two *loxP* sequences at desired timings. We constructed *E. coli* cell strains that harbor the PA-Cre system and the photocleavable fluorescently labeled chloramphenicol-resistance gene (Koganezawa et al., 2022). We observed the *E. coli* cells at the single-cell level in the microfluidic device and deleted the genes directly in the device by blue light illumination.

The duration of blue light illumination significantly influences the fraction of gene-deleted cells in the population. Therefore, by adjusting the illumination duration, we can simultaneously observe the dynamics of gene-deleted cells and non-deleted cells in a cell population. Since the target chloramphenicol resistance gene is tagged with the gene for the fluorescent protein mCherry, the fractions of gene-deleted cells are measurable simply by fluorescent microscopy. Here, we describe the protocol for measuring the fraction of gene-deleted cells based on batch-culture assay.

## Materials and reagents

1. Glass test tube 16.5φ × 165L (IWAKI, catalog number: B14-002-160)
2. Molton cap (AS ONE, catalog number: 6-352-07)
3. 1.5 mL microtube (TreffLab, catalog number: 96.07246.9.01)
4. Minisart^®^ syringe filter (Sartorius, catalog number: S7597-FXOSK)
5. Sterilized petri dish φ90 × 15 mm (AS ONE, catalog number: 1-7484-01)
6. Aluminum foil
7. *E. coli* strain, carrying a fluorescently tagged target gene flanked with two *loxP* sequences and photoactivatable Cre (PA-Cre) system. Here, we used YK0083, which has a chloramphenicol resistance gene, *cat*, as a target gene for demonstration. The mCherry-tagged *cat* gene sandwiched with two *loxP* sequences was introduced into the *intC* locus on the genome. The genes for the PA-Cre system, creN-nmag and pmag-creC, are placed downstream of P*_LlacO1_* promoter on a plasmid. These genes are inducible by IPTG. This strain is available by contacting the authors.
8. Difco^TM^ LB broth, Miller (Luria-Bertani) (BD, catalog number: 244620)
9. Agar (Wako, catalog number: 010-15815)
10. Difco^TM^ M9 minimal salts, 5× (BD, catalog number: 248510)
11. D(+)-glucose (Wako, catalog number: 049-31165)
12. MgSO_4_·7H_2_O (Wako, catalog number: 131-00405)
13. CaCl_2_·2H_2_O (Wako, catalog number: 31-00435)
14. MEM amino acids solution (50×) (SIGMA, catalog number: M5550)
15. Ampicillin sodium (Wako, catalog number: 016-23301)
16. Isopropyl-β-D(-)-thiogalactopyranoside (IPTG) (Wako, catalog number: 094-05144)
17. Elix^®^ water produced by Milli-Q^®^ Integral 3 (Merck, catalog number: ZRXQ003T0)
18. Milli-Q^®^ water produced by Milli-Q^®^ Integral 3 (Merck, catalog number: ZRXQ003T0)
19. LB broth (see Recipes)
20. LB agar containing 100 μg/mL of ampicillin (Amp) (see Recipes)
21. M9 medium with 0.2% glucose and amino acids (see Recipes)
22. 1 M IPTG (see Recipes)
23. 50 mg/mL Amp (see Recipes)

## Equipment

1. Centrifuge (Hitachi, himac, CT 15RE)
2. Spectrometer (Shimadzu, UV-1800)
3. Blue light illuminator (Power Supply: CCS Inc., ISC-201-2; Blue light Illuminator: CCS Inc., SLM-150X150-BB)
4. BioShaker (TAITEC, BR-21FP)
5. Incubator (MITSUBISHI ELECTRIC ENGINEERING, SLC-25A)
6. Stereomicroscope (Stereomicroscope: Olympus, SZ61; LED source: NIGHTSEA, SFA-GR)
7. Portable photodiode-based laser power meter (Gentec-EO, PRONTO-Si)

## Procedure

**Figure 2.**
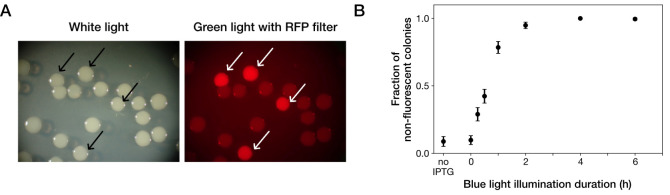
Photoactivatable Cre-mediated gene deletion. (A) Stereomicroscopic pictures of YK0083 colonies after blue-light illumination under ambient light (left) and excitation light for detecting mCherry fluorescence (right). Cells were exposed to blue light for 1 h. Arrows show colonies generated from cells from which target genes were not deleted with blue-light illumination. (B) Blue-light illumination duration dependency of gene deletion efficiency. In case of less than 6 h blue-light illumination, test tubes were covered with aluminum foil and shaken at 37 °C, so that the time from the start of blue-light illumination to spreading on the LB agar was 6 h and consistent among the conditions. “no IPTG” indicates the condition where cells were cultured without IPTG and not exposed to blue light. Black points indicate the means. Error bars indicate standard errors (N = 239 for no IPTG, N = 327 for 0 h, N = 335 for 0.25 h, N = 373 for 0.5 h, N = 344 for 1 h, N = 350 for 2 h, N = 296 for 4 h, and N = 209 for 6 h). Reprinted/adapted from Koganezawa et al. (2022).

- **Sample preparation**
  1. Culture *E. coli* cells in LB broth with 50 μg/mL of Amp at 37 °C with shaking overnight.
  2. Centrifuge 100 μL of the overnight culture at 21,500× *g* for 1 min at room temperature.
  3. Remove the supernatant.
  4. Resuspend the cellular pellet in 1 mL of M9 medium with 0.2% glucose and amino acids.
  5. Measure the OD_600_ of the resuspended cells.
  6. Adjust the OD_600_ to 0.001 in 2 mL of M9 medium with 0.2% glucose and amino acids containing 50 μg/mL of Amp and 0.1 mM IPTG.
  7. Cover the test tube with the aluminum foil.
  8. Cultivate the cells at 37 °C with shaking for 3 h.
- **Gene deletion induction**
  1. Remove the aluminum foil after the cultivation.
  2. Expose the test tube to blue light using blue-light illuminator (light intensity: 6.8 mW) for the desired duration (Figures 1 and 2, see Notes).
    **Figure 1.**
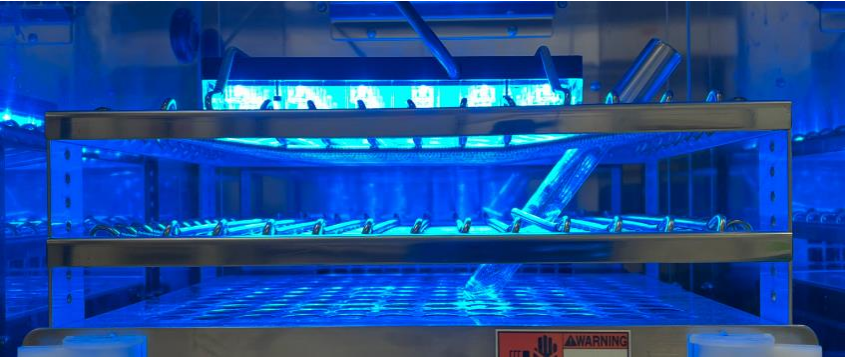
Blue-light illumination inside a bio shaker with an illuminator. A blue-light illuminator was directly fastened onto a test tube spring holder inside a bio shaker. It casts a blue light downward on *E. coli* cultures. Light intensity was adjusted to 6.8 mW, measured with a laser power meter (λ = 470 nm).
- **Calculation of gene-deleted cells in batch**
  1. Dilute the cultures exposed to blue light to OD_600_ = 1.0 × 10^-6^, which is equivalent to approximately 7 × 10^2^ cells/mL.
  2. Spread 150 μL of the diluted cultures on LB agar plate containing 100 μg/mL of Amp.
  3. Cover the agar plates with aluminum foil.
  4. Incubate the plates at 37 °C for 18 h.
  5. Count the number of colonies under ambient light and excitation light for examining mCherry fluorescence using a stereomicroscope (Figure 2A).
  6. Calculate the fraction of gene-deleted cells as the number of non-fluorescent colonies divided by the number of total colonies.

## Notes

The fraction of gene-deleted cells varies depending on the duration of blue-light illumination (Figure 2B). Therefore, an experimenter should determine the duration of blue-light exposure depending on the purpose of the experiment.

Cre-mediated gene deletion with blue-light illumination is detectable with loss of mCherry fluorescent signal.

## Recipes

1. **LB broth**
  - Mix the following:
    25 g of Difco^TM^ LB broth, Miller (Luria-Bertani)
    1 L of Elix^®^ water
  - Autoclave at 121 °C for 15 min.
  - Store at room temperature.
  - At the time of use, add 50 mg/mL Amp on a clean bench as appropriate, if needed.
2. **LB agar containing 100 μg/mL of Amp**
  - Mix the following:
    25 g of Difco^TM^ LB broth, Miller (Luria-Bertani)
    15 g of agar
    1 L of Elix^®^ water
  - Autoclave at 121 °C for 15 min.
  - Add 2 mL of 50 mg/mL Amp on a clean bench.
  - Dispense 25 mL in each 90 mm dish on a clean bench.
  - Store at 4 °C.
3. **M9 medium with 0.2% glucose and amino acids**
  - Prepare the following reagents:
**5× M9**
    1) Dissolve 56.4 g of Difco^TM^ M9 minimal salts, 5× in 1 L of Milli-Q^®^ water.
    2) Autoclave at 121 °C for 15 min.
    3) Store at room temperature.
**20% glucose**
    1) Dissolve 10 g of D(+)-glucose in Milli-Q^®^ water to 50 mL.
    2) Sterilize using a 0.2 μm pore filter.
    3) Store at 4 °C.
**1 M MgSO_4_**
    1) Dissolve 12.3 g of MgSO_4_·7H_2_O in Milli-Q^®^ water to 50 mL.
    2) Sterilize using a 0.2 μm pore filter.
    3) Store at 4 °C.
**1 M CaCl_2_**
    1) Dissolve 7.4 g of CaCl_2_·2H_2_O in Milli-Q^®^ water to 50 mL.
    2) Sterilize using a 0.2 μm pore filter.
    3) Store at 4 °C.
**Sterilized water**
    1) Autoclave Milli-Q^®^ water at 121 °C for 15 min.
    2) Store at room temperature.
  - Mix the following on a clean bench:
    200 mL of 5× M9
    10 mL of 20% glucose
    10 mL of MEM amino acids solution (50×)
    2 mL of 1 M MgSO_4_
    100 μL of 1 M CaCl_2_
    778 mL of sterilized water
  - Store at 4 °C.
  - At the time of use, add antibiotics or IPTG on a clean bench as appropriate, if needed.
4. **1 M IPTG**
  - Dissolve 2.383 g of IPTG in Milli-Q^®^ water to 10 mL.
  - Sterilize using a 0.2 μm pore filter.
  - Dispense in microtubes on a clean bench.
  - Store dispensed tubes at -20 °C.
5. **50 mg/mL Amp**
  - Dissolve 500 mg of ampicillin sodium in 10 mL of Milli-Q^®^ water.
  - Sterilize using a 0.2 μm pore filter.
  - Dispense in microtubes on a clean bench.
  - Store dispensed tubes at -20 °C.
